# Anti-inflammatory effect of *Salsola komarovii* extract with dissociated glucocorticoid activity

**DOI:** 10.1186/s12906-020-02979-4

**Published:** 2020-06-05

**Authors:** Ji Hyun Seo, Mu Hyun Jin, Yun Hee Chang

**Affiliations:** LG Science Research Park, LG Household and Healthcare Ltd., 70, Magokjoongang 10-ro, Gangseo-gu, Seoul, 07795 Korea

**Keywords:** *Salsola komarovii*, Glucocorticoid, Dissociated glucocorticoid, Anti-inflammation

## Abstract

**Background:**

Glucocorticoids (GCs) are anti-inflammatory drugs widely used to treat acute and chronic inflammatory diseases. However, despite their excellent efficacy, the long-term use of GCs is relatively limited owing to their adverse effects. Recent studies have sought to reduce these adverse effects by developing dissociated GCs that bind to GC receptors (GRs) to induce potent anti-inflammatory effects without the transcription of GC response element (GRE)-promoted genes. Some species of the genus *Salsola* are used in traditional Chinese medicine to treat cancer, hypertension, and inflammation. In this study, we investigated the potential dissociated GC activities and underlying mechanisms of *Salsola komarovii* (SK), which is native to Korea.

**Methods:**

To determine whether SK ethanol extract (SEE) directly interacts with the GR, an in vitro fluorescence polarization based-GR competitor assay was performed. The effect of SEE on the transcriptional activity of nuclear factor (NF)-κB and GRE was confirmed in HepG2 cells using the Cignal reporter assay. The anti-inflammatory effect of SK was determined by assessing lipopolysaccharide (LPS)-induced interleukin (IL)-6 production. To confirm whether SEE induces GRE-driven gene expression, preadipocyte differentiation followed by lipid deposition was performed in the presence of SEE.

**Results:**

SEE exhibited GR binding activity in the fluorescence polarization competitive binding assay and induced GR nuclear translocation. It also interfered with the nuclear translocation of NF-κB and the NF-κB-dependent transcriptional activity based on the immunofluorescence analysis and reporter assay, respectively. SEE exerted anti-inflammatory effects by reducing LPS-induced IL-6 production as effectively as hydrocortisone (positive control). SK did not induce GRE-driven gene expression and preadipocyte differentiation, which is one of the major adverse effects of GCs.

**Conclusions:**

Collectively, these results suggest that SK could be a novel and safe anti-inflammatory agent with dissociated GC properties and, therefore, it has great potential for use in treating inflammatory disorders.

## Background

Globally, numerous people experience inflammatory disorders, such as asthma, rheumatoid arthritis, psoriasis, and serious auto-immune and auto-inflammatory diseases [1]. Glucocorticoids (GCs) are commonly used for the treatment of numerous inflammatory disorders because of their effective whole-body anti-inflammatory activity [2, 3]. However, the chronic use of GCs causes undesirable adverse effects such as skin atrophy, wound healing inhibition, osteoporosis, obesity, hyperglycemia, and glaucoma [4, 5]. Therefore, GCs, as anti-inflammatory agents, act as a double-edged sword.

In the absence of GCs, GC receptors (GRs) are predominantly found in the cytoplasm as complexes with accessory proteins such as heat shock protein (hsp) 70 and hsp 90 [6]. When GCs bind to the GRs in the cytoplasm, the GC-bound GRs are transported along the microtubules into the nucleus [7]. The GR represses the production of inflammatory cytokines such as IL-1α, IL-1β, and IL-6 by tethering to the nuclear transcription factor, nuclear factor (NF)-κB, in a process called “transrepression.” NF-κB is involved in multiple immune and inflammatory responses via the regulation of immune cells and pro-inflammatory gene expression [8]. In addition, NF-κB regulates neural progenitor cell maintenance and inhibits neuronal differentiation, which are major mechanisms in neurodevelopment [9]. In cells, NF-κB bound to inhibitory proteins such as the IκB family members is normally sequestered into the cytoplasm. Immediately after activation by certain signals, NF-κB is translocated into the nucleus, where it exerts its roles. Therefore, GCs, as immune suppressive drugs that do not regulate NF-κB, have been used to treat inflammatory disorders [10] because GC-bound GR suppresses inflammatory responses by preventing NF-κB from transcribing pro-inflammatory genes, the process is called tethering. The GRs also induce severe adverse effects by directly binding to inducible enhancer elements, GC response elements (GREs), in a process called “transactivation” [11]. Therefore, considerable efforts have been made to develop dissociated GCs, which induce GR-dependent transrepression without stimulating GR transactivation [12–17]. These efforts are based on the premise that the efficacy of dissociated GCs will be the same or better than that of classic GCs with minimal adverse effects [18, 19]. The compound 2-(4-acetoxyphenyl)-2-chloro-N-methyl-ethylammonium chloride is a stable analog of the hydroxy phenyl aziridine precursor found in *Salsola tuberculatiformis* and ginsenoside Rg1 is the most abundant and well-known triterpene saponin in *Panax ginseng*. Recently, these compounds were found to exhibit dissociated GC activity with reduced adverse effects [13, 14]. However, there are only a few studies on plant-derived anti-inflammatory agents with dissociated GC activity.

The genus *Salsola* consists of 200 species and a few members, such as *S. baryosoma*, *S. foetida*, *S. richteri*, and *S. tragus*, have been used in Chinese traditional medicine for the treatment of cancer, hypertension, and inflammation [20–22]. *Salsola komarovii* (SK) grows in sand dunes and beaches in Japan, China, and Korea. The plant, an annual herbaceous halophyte, is used for the rehabilitation and reclamation of degraded saline lands [23]. SK has been traditionally used to treat hyperpyrexia, hypertension, inflammation, jaundice, and gastrointestinal diseases [24]. Recently, several antioxidant constituents in SK, including 7 flavonoids and 2 phenolic amides, have been identified by high-performance liquid chromatography–mass spectrometry [25]. A phytochemical analysis of SK revealed 19 compounds, namely, 5 lignan glycosides, 7 megastigmane glycosides, and 7 phenolic compounds, and among them, 3 compounds, namely, alangilignoside C, conicaoside, and blumenyl B β-D-glucopyranoside, induced the secretion of neural growth factor (NGF) [26]. In addition, the ameliorating effect of SK against gastritis and gastric ulcers was verified in vivo in a mouse model [24]. However, other biological activities of SK have been rarely studied.

The clinical use of classic GCs has been limited because of their debilitating adverse effects. Thus, a comprehensive understanding of GR signaling pathways would help develop strategies to overcome the limitations of classic GCs. Consequently, dissociated GCs, which present an ideal balance between therapeutic advantages and adverse effects, have been studied [19, 27]. In this study, we investigated the novel dissociative GC activity of SK ethanol extract (SEE) using an in vitro cell model and compared it with that of a standard GC, hydrocortisone (HC). We further elucidated the potential dissociated GC mechanisms of SEE by analyzing relevant signaling pathways and factors.

## Methods

### Chemicals and reagents

HC (#H0888), human tumor necrosis factor-α (TNF-α, #8916), lipopolysaccharide (LPS, #L4391) from *Escherichia coli* O111:B4, phorbol 12-myristate 13-acetate (PMA, #P8139), insulin (#I3536), isopropanol (#9516), and 3-isobutyl-1-methylxanthine (IBMX, #5879) were purchased from Sigma-Aldrich (St. Louis, MO, USA). Dulbecco’s phosphate-buffered saline (DPBS) without calcium and magnesium (#14190144) was purchased from Gibco (Thermo Fisher Scientific, Waltham, MA, USA). Cignal reporter assay kits for the NF-κB (#CCS-013 L) and GRE (#CCS-006 L) pathways were purchased from Qiagen (Hilden, Germany) and the DualGlo luciferase reporter system kit (#E1960) was procured from Promega (Madison, WI, USA). Genjet™ in vitro DNA transfection reagent (Ver. II, #SL100489) was purchased from (Rockville, MD, USA).

Polyclonal rabbit anti-human glucocorticoid receptor (#ab3578, 1 mg/mL; Abcam, Cambridge, UK) and monoclonal rabbit anti-human NF-κB p65 (#C22B4; Cell Signaling Technology, Danvers, MA, USA) antibodies were used for the immunofluorescence assay. Alexa 594 goat anti-rabbit antibody (#ab150080, Abcam) was used as the secondary antibody and 4′,6-diamidino-2-phenylindole dihydrochloride (DAPI, #D9542; Sigma-Aldrich) was used to counter-stain DNA.

### SK ethanol extract preparation

SEE was prepared as previously described [24]. Air-dried powdered SK was purchased from Yongmoon Farm (Yongin, Korea); 100 g of the powder was extracted with 2000 mL of 50% ethanol (v/v) at 80 °C under reflux conditions for 3 h. The extract was then filtered using qualitative filter paper (#1004090; Whatman, Maidstone, UK) under decompression conditions. The ethanol extract (28.5 g) was vacuum-dried using a rotary evaporator (Buchi, Flawil, Switzerland); the extract yield was 28.5%. The dry ethanol extract was dissolved in distilled water to a concentration of 20% (w/v) for use in the in vitro tests.

### Cell culture

For the immunofluorescence assay, HaCaT human keratinocyte cell line was obtained from AddexBio (San Diego, CA, USA) and cultured in Dulbecco’s modified Eagle’s medium (DMEM, #11995073) supplemented with 10% fetal bovine serum (FBS, #16000044) and 1% antibiotic-antimycotic (#15240062; all from Gibco, Thermo Fisher Scientific). For transfection, HepG2 human liver carcinoma cell line was purchased from the Korean Cell Line Bank (Seoul, Korea) and cultured in Roswell Park Memorial Institute 1640 medium (#11875–093; Gibco) supplemented with 10% FBS and 1% antibiotic-antimycotic. For the IL-6 enzyme-linked immunosorbent assay (ELISA), Thp-1 human monocytic cell line was purchased from the American Type Culture Collection (Manassas, VA, USA) and cultured in Roswell Park Memorial Institute 1640 with 25 mM HEPES (#22400–089; Gibco) supplemented with 10% FBS, 1% antibiotic-antimycotic, and 0.1% 2-mercaptoethanol (#21985–023; Gibco). For adipogenesis, 3 T3-L1 preadipocytes were obtained from the American Type Culture Collection and maintained in DMEM supplemented with 10% bovine calf serum (BCS, #16170078; Gibco) and 1% antibiotic-antimycotic. All cell lines were maintained at 37 °C in a humidified atmosphere with 5% CO_2_.

### Fluorescence polarization GR competitor assay

The PolarScreen™ GR competitor assay red kit (PerkinElmer, Waltham, MA, USA) was used according to the manufacturer’s instructions. Briefly, the full-length GR and fluormone GS red were used at the recommended concentrations of 1 mg/mL and 1.4 nM, respectively. Ten microliters of a mixture of SEE or HC (5 μL) and GS screening buffers (5 μL) was added to black and low-volume 384-well microplates (#CLS3824; Sigma-Aldrich). Then, 4 × fluormone (5 μL) followed by 4 × GR (5 μL) was added into each well, and SEE or HC at each concentration was tested in triplicate. After 3 h of incubation 24 °C, fluorescence polarization (FP) was measured using the 2103 EnVision multi-label plate reader (PerkinElmer) at 531 nm excitation and 595 nm emission wavelengths. The FP values were plotted against the sample concentration and the data were analyzed using GraphPad Prism™ software 8 (GraphPad Software, Inc., San Diego, CA, USA).

### Immunofluorescence analysis

For visualization of NF-κB, HaCaT cells (2 × 10^4^ cells/well) were seeded in 24-well culture plates and incubated for 24 h. The cells were then treated with medium containing 1 μM HC or 40 μg/mL SEE for 20 h, followed by stimulation with 10 ng/mL TNF-α for 4 h. To visualize the GR, HaCaT cells were similarly treated for 24 h under the same conditions except without TNF-α stimulation. The cells were then stained with a monoclonal antibody against NF-κB (1:100) or polyclonal antibody against GR (1:20), followed by the secondary goat anti-rabbit Alexa 594 antibody (1:1000). The cell nuclei were then counter stained with 2.5 μg/mL DAPI and the cells were mounted in DPBS medium. Immunofluorescence images were acquired using the EVOS™ FL Auto2 imaging system (Thermo Fisher Scientific).

### Transient transfection and reporter assay

The effect of SEE on the transcriptional activity of NF-κB and GRE was confirmed using the Cignal reporter assay kits (Qiagen) consisting of NF-κB-responsive firefly luciferase reporter (#CCS-013 L) and GR-responsive firefly luciferase reporter (#CCS-006 L), respectively, and a Renilla construct luciferase reporter (40:1). HepG2 cells (2 × 10^5^ cells/well) were seeded in 24-well culture plates, incubated for 24 h, and then transfected with 200 ng of plasmid DNA using 3 μL of Genjet™ in vitro DNA transfection reagent according to the manufacturer’s instructions.

After 18 h of transfection, the cells were treated with medium containing HC (1 μM) or SEE (1000 and 5000 μg/mL) for 30 min, followed by stimulation with 10 ng/mL TNF-α for 5 h for the NF-κB pathway analysis or medium containing 1 μM HC or SEE (1000, 2500, and 5000 μg/mL) for 6 h for the GRE pathway analysis. The expression of the luciferase reporter gene was analyzed using the DualGlo luciferase reporter system kit. Luminescence was measured using the Wallac Victor3 1420 multilabel counter (PerkinElmer, MA, USA). The level of firefly luciferase activity was normalized to that of Renilla luciferase in each experiment.

### Quantitative measurement of IL-6 production

To measure the levels of the pro-inflammatory cytokine IL-6, THP-1 cells (2.5 × 10^5^ cells/well) were seeded in 24-well culture plates, grown for 24 h, and then treated with PMA at a concentration of 50 ng/mL to induce differentiation. After 24 h of incubation, the medium was replaced with fresh medium containing HC (1 μM) or SEE (8, 40, and 200 μg/mL) with 1 μg/mL LPS for stimulation. After 24 h, the culture supernatants of the differentiated THP-1 cells were harvested and IL-6 concentration was measured using the human IL-6 Duoset ELISA kit (R&D Systems, Minneapolis, MN, USA) and Epoch microplate spectrophotometer (Biotek Industries, Winooski, VT, USA) according to the manufacturer’s instructions.

### Preadipocyte differentiation and Oil Red O staining

3 T3-L1 preadipocytes (2 × 10^4^ cells/well) were seeded and grown in DMEM supplemented with 10% BCS for 24 h in 24-well culture plates. To induce adipogenesis, the cells were treated with 10 μg/mL insulin and 0.5 mM IBMX in the presence of HC (1 μM) or SEE (8, 40, and 200 μg/mL) in DMEM with 10% FBS for 4 days. The medium was replaced every 2 day with fresh medium containing the same components as the previous differentiation medium except for IBMX for 4 days. After differentiation, the cells were washed with DPBS and fixed with 4% formaldehyde in DPBS for 60 min, and then stained with filtered Oil Red O solution (0.5% in 60% isopropanol) for 60 min at 24 °C. The cells were then washed twice with distilled water to remove the unbound dye and photographed. Oil Red O stain was extracted with isopropanol and the absorbance of the extracted solution was measured at 520 nm using the Epoch microplate spectrophotometer.

### Statistical analysis

**Fig. 1:**
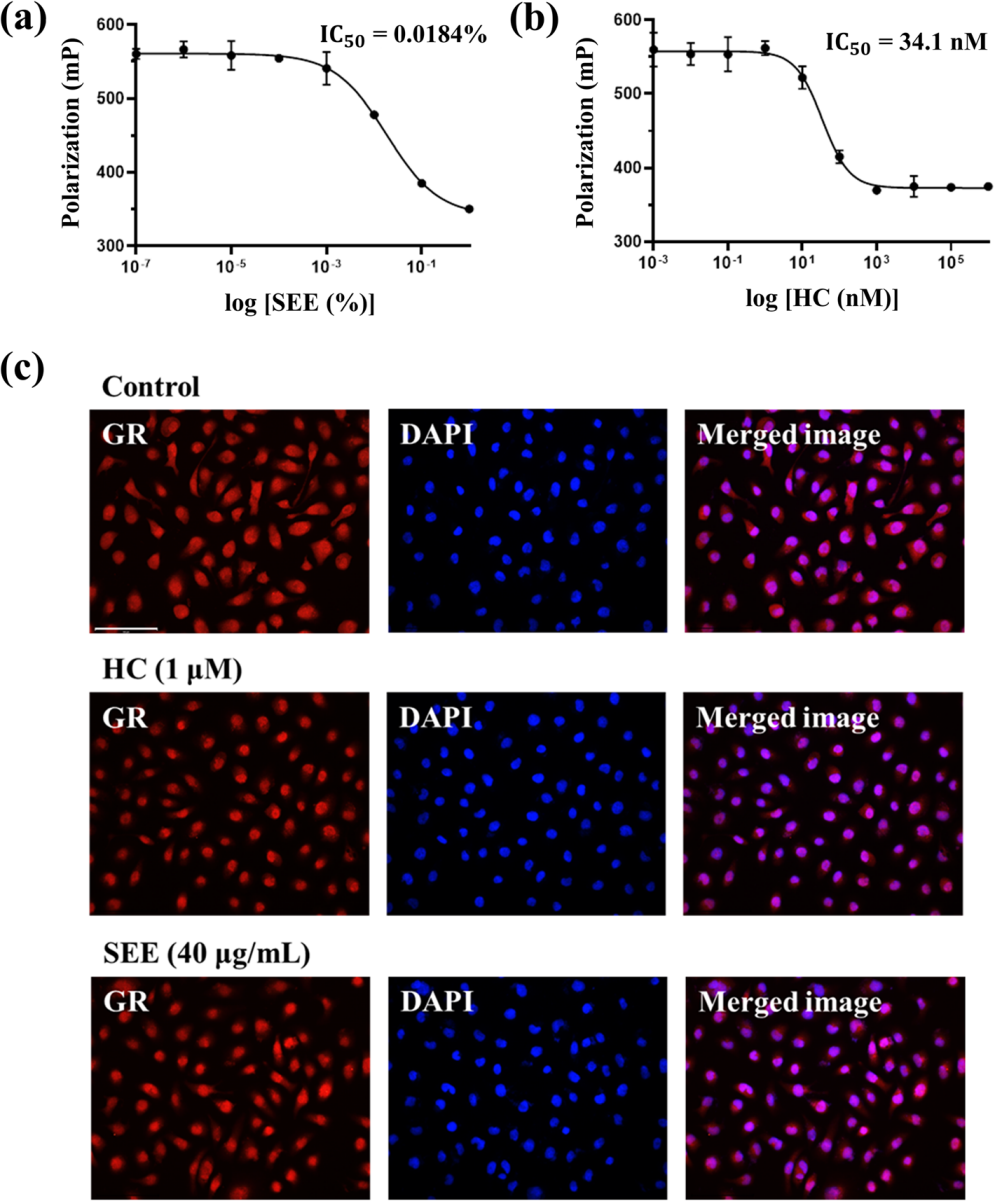
*Salsola komarovii* ethanol extract (SEE) as a glucocorticoid receptor (GR) ligand induces nuclear GR translocation. Binding affinity of **a** SEE and **b** hydrocortisone (HC) to the GR. Data are shown as fluorescence polarization (mP). The results are presented as mean ± standard error of the mean (SEM) of three independent experiments. **c** Immunofluorescence analysis of GR translocation into nucleus by HC or SEE. HaCaT cells were incubated for 24 h with 1 μM HC (positive control) or 40 μg/mL SEE followed by staining of GR (red fluorescence) and nucleus with 4′,6-diamidino-2-phenylindole dihydrochloride (DAPI, blue fluorescence); original magnification 400×; scale bar = 100 μm

**Fig. 2:**
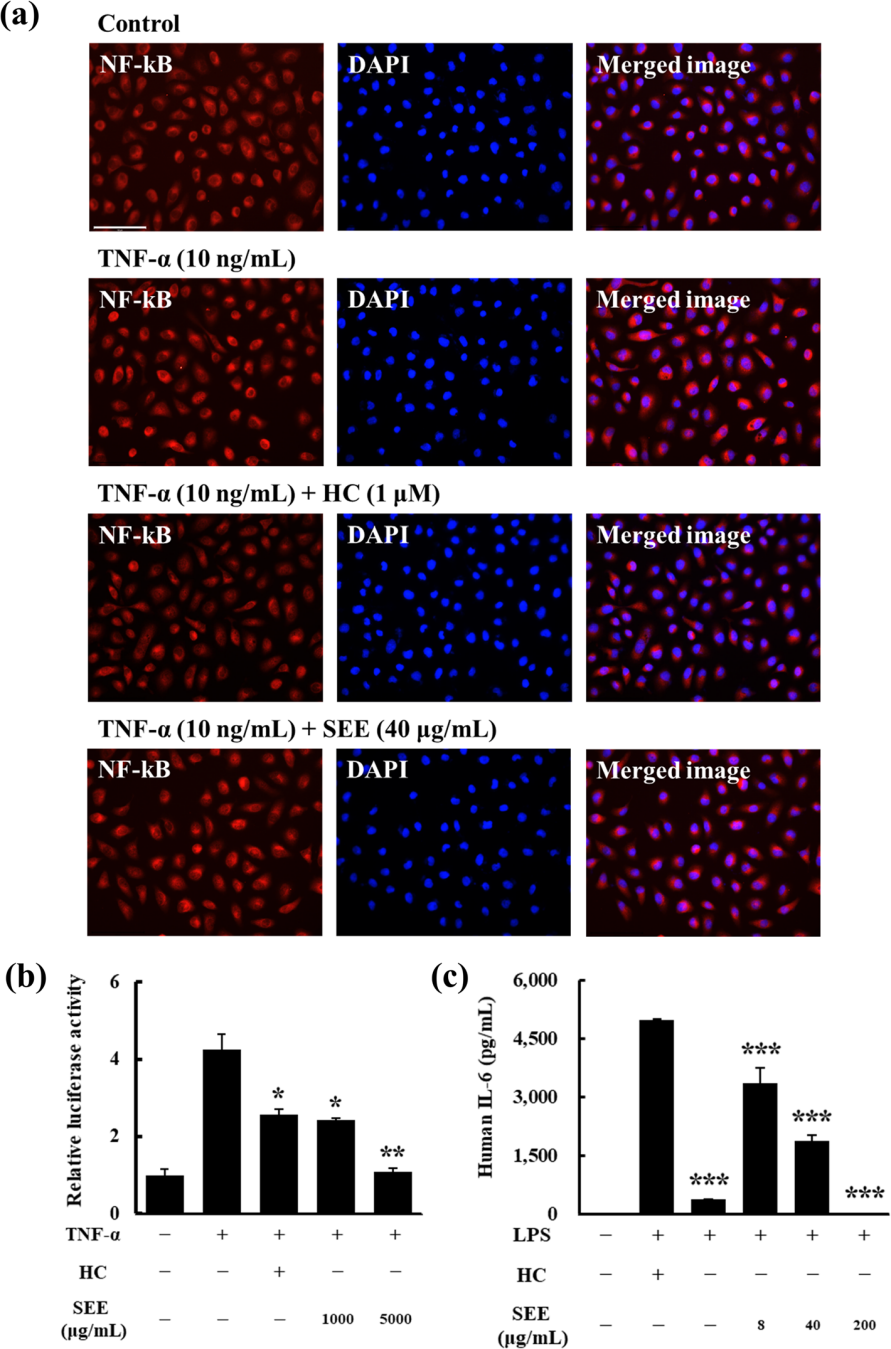
*Salsola komarovii* ethanol extract (SEE) attenuates NF-κB activation and cytokine production. The untreated group was used as the control. **a** Immunofluorescence analysis of NF-κB translocation into the nucleus in HaCaT cells. The cells were pre-treated with 1 μM HC (positive control) or 40 μg/mL SEE before 10 ng/mL TNF-α stimulation, followed by staining of NF-κB (red fluorescence) and nucleus with DAPI (blue fluorescence). Original magnification, 400×; scale bar = 100 μm. **b** Effect of SEE on NF-κB activation induced by TNF-α treatment, determined using the luciferase reporter gene assay. After being transiently transfected with the NF-κB-responsive reporter and Renilla plasmid, HepG2 cells were pre-treated with 1 μM HC or SEE (1000 or 5000 μg/mL) before 10 ng/mL TNF-α stimulation. Luciferase activity was normalized to Renilla plasmid luminescence. Data are shown as relative luciferase activity corrected using that of the control group. The results are presented as mean ± standard error of the mean (SEM) of three independent experiments. *p < 0.05, **p < 0.01 versus TNF-α-treated group. **c** Effect of SEE on LPS-induced pro-inflammatory cytokine production. IL-6 ELISA was performed using the culture supernatant of Thp-1 cells, which were treated with 1 μM HC or SEE (8–200 μg/mL) as well as 1 μg/mL LPS . Data are presented as mean ± SEM of three independent experiments. ***p < 0.001 vs. LPS-treated group

**Fig. 3:**
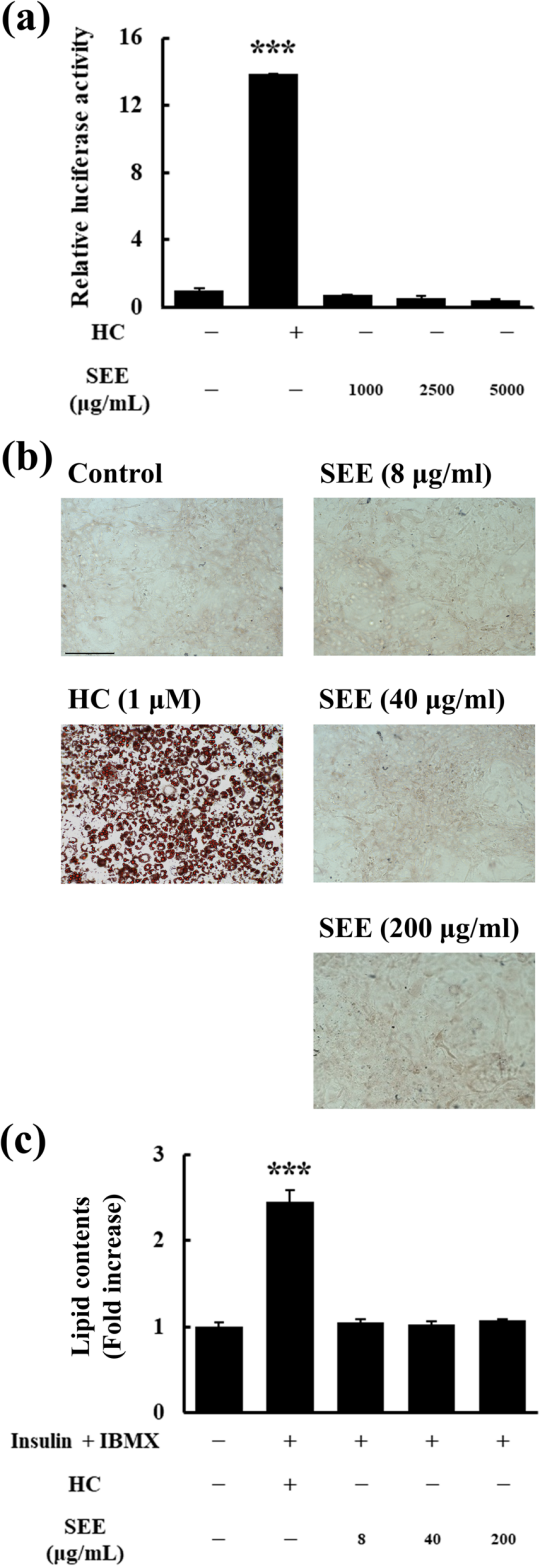
*Salsola komarovii* ethanol extract (SEE) does not induce glucocorticoid response element (GRE)-driven adipogenesis. **a** Effect of SEE on GRE induction, determined using the luciferase reporter gene assay. HepG2 cells were transiently transfected with the Cignal GRE reporter reagent including the GRE-containing reporter and Renilla plasmid. The transfection process and data analysis were similar to those of the NF-κB-driven luciferase reporter gene assay in Fig. 2b. The HC-treated group was used as the positive control. ***p < 0.001 vs. untreated group. **b** and **c** Effect of SEE on lipid accumulation in 3 T3-L1 cells. **b** The preadipocytes were differentiated in the absence or presence of the SEE or HC for 8 days, and then stained. Oil Red O-stained cells were photographed and the representative microscopic images of three independent experiments are presented. Original magnification 200×; scale bar = 200 μm. **c** Lipid content in Oil Red O-stained adipocytes was quantified using the absorbance measured at 520 nm. The results are presented as mean ± standard error of the mean (SEM) of three independent experiments. ***p < 0.001 vs. untreated group

Data in all figures except microscopic images are expressed as mean ± standard error of the mean (SEM) of three independent experiments. Statistical comparisons between two groups (Figs. 2b and c, 3a and c) were performed using a one-tailed, unpaired Student’s *t*-test. Values with *p* ≤ 0.05 were considered statistically significant. Regression analysis was used to estimate the half-maximal inhibitory concentration (IC_50_) from the polarization plots (Fig. 1a and b).

## Results

### SEE activates the GR as a ligand

To investigate whether SEE directly interacts with the GR, an in vitro fluorescence polarization based-GR competitor assay was performed (Fig. 1a and b). SEE competed with the fluormone GS for binding to the full-length GR (IC_50_ = 0.01839%, Fig. 1a). However, SEE at concentrations of > 1% could not be analyzed because of the color of SEE. HC was used as the positive control in the GR binding assay (IC_50_ = 34.1 nM, Fig. 1b).

Next, we verified whether SEE binding induces the translocation of GR into the nucleus of HaCaT cells by visualizing the cellular location of GR using the immunofluorescence assay (Fig. 1c). GR (red fluorescence) was detected in the whole region of control cells, whereas in cells treated with 1 μM HC for 24 h, the GR and nuclear region (blue fluorescence) staining almost completely overlapped. After treatment with 40 μg/mL SEE, the GR significantly translocated from the cytoplasm to the nucleus, indicating that SEE bound to the GR to induce its cytoplasmic activation and the subsequent translocation to the nucleus.

### Anti-inflammatory properties of SEE are mediated via the GR-mediated NF-κB pathway

To determine that the anti-inflammatory effect of SK is mediated by GR binding and its nuclear translocation, we visualized the location of NF-κB by immunofluorescence in HaCaT cells (Fig. 2a). After stimulating HaCaT cells with 10 ng/mL TNF-α, NF-κB was activated and translocated to the nucleus, whereas in the control, it was mainly localized in the cytoplasm. Although 1 μM HC exhibited a stronger inhibitory effect against NF-κB translocation than 40 μg/mL SEE, pre-treatment with SEE before TNF-α stimulation significantly prevented NF-κB nuclear translocation.

Next, a NF-κB reporter plasmid was transiently transfected into HepG2 cells to confirm whether SEE blocks NF-κB-driven pro-inflammatory gene expression (the so-called tethering transrepression, Fig. 2b). TNF-α induced an approximately 4-fold increase in gene transcription compared with the control, whereas pre-treatment with 1000 μg/mL SEE reduced the levels close to that with 1 μM HC pre-treatment. Furthermore, pre-treatment with 5000 μg/mL SEE blocked the transcription level to near basal values.

Lastly, the production of the NF-κB-driven pro-inflammatory cytokine, IL-6, was measured in Thp-1 cells (Fig. 2c). After LPS stimulation, the IL-6 concentration in the supernatant was increased by approximately 5000 pg/mL compared with the untreated control, which did not produce IL-6. In contrast, pre-treatment with SEE (8–200 μg/mL) reduced the production of IL-6 in a dose-dependent manner. Especially, 200 μg/mL SEE blocked LPS-induced IL-6 production to the basal level of the untreated control.

### SEE does not induce GRE-driven adipogenesis

To confirm whether SEE induces GRE-driven gene expression, a GRE-driven luciferase reporter plasmid was transiently transfected into HepG2 cells, and the cells were then treated with SEE (1000–5000 μg/mL) or 1 μM HC (Fig. 3a). The results showed that SEE barely induced luciferase expression. Moreover, compared with the control treatment, HC increased GRE-driven luciferase expression by approximately 14-fold. This observation is indicative of the dissociated GC activity of SEE.

Next, we investigated whether SEE induces the adipogenesis of 3 T3-L1 preadipocytes. The adipogenesis was confirmed on day 8 of the experiment by lipid droplet accumulation in the cells visualized by Oil Red O staining. The treatment of 3 T3-L1 cells with SEE (8–200 μg/mL) did not promote preadipocyte differentiation, whereas 1 μM HC induced complete differentiation of preadipocytes as expected on day 8 (Fig. 3b). The optical density of the Oil Red O-eluted solution was two-fold higher in 3 T3-L1 cells treated with 1 μM HC than that in untreated cells (Fig. 3c). These results indicated that SEE did not promote adiopogenesis and could not likely induce obesity associated with hypertrophy of adipocytes.

## Discussion

GR binding is a prerequisite for the GR ligand activity [28]. Inactive GRs remain in the cytoplasm because they bind to the accessory proteins such as hsp 90 and hsp 70 [29]. However, GRs can be activated by binding to GR-specific ligands, leading to their release from the accessory proteins; then, the activated ligand-GR complex is translocated to the nucleus. First, our study demonstrated the binding of SEE to the GRs based on the measurement of the FP of GR. The extent of GR-ligand binding can be quantified by measuring the extent of depolarization, which indicates the difference in FP according to the physical principles of FP. Although SEE presented a lower binding affinity than HC, this result suggests that some components of SK bind to the GR in a manner similar to that of HC. Furthermore, the immunofluorescence analysis confirmed that SEE induced the activation and translocation of GR to the nucleus.

The activation of the ligand-GR complex suppresses inflammatory responses by preventing the transcription factor NF-κB from transcribing pro-inflammatory genes such as IL-6 and IL-8. The inhibitory mechanism of the complex can be divided into two categories based on the location of the ligand-GR complex. In one mechanism, activated cytoplasmic GR inhibits the translocation of NF-κB to the nucleus by blocking the degradation of the inhibitor of NF-κB (Iκ-B) [30], whereas in the other mechanism, activated nuclear GR prevents the transcription of pro-inflammatory genes by tethering to NF-κB [31]. Therefore, we verified that SEE attenuated cytoplasmic NF-κB translocation to the nucleus using the immunofluorescence analysis and that SEE significantly repressed TNF-α-induced NF-κB-dependent luciferase expression in HepG2 cells and LPS-driven inflammatory cytokine production in Thp-1 cells.

GRE-driven adipogenesis, which is mediated by the adipogenic master switch, peroxisome proliferator-activated receptor-γ, is one of the prominent GC adverse effects [32]. Although the underlying mechanism of the effect of GCs on lipid accumulation has not been completely elucidated, the nuclear GR-ligand complex has been demonstrated to bind to GRE (transactivation), which then mediates adipogenesis, followed by body fat deposition [10, 29]. Therefore, to minimize the adverse effects of GC, transactivation and the subsequent GRE-driven gene expression should be inhibited. We verified that the SEE-bound GR did not bind to GRE using the GRE-driven reporter gene assay in HepG2 cells and that it did not stimulate GRE-driven adipogenesis in 3 T3-L1 cells.

Although the concept of dissociated GC is still under debate, dissociated GCs have been developed to replace classic GCs because their anti-inflammatory activities are associated with a low probability of inducing adverse effects [33]. To date, little is known about traditional medicinal plants with dissociated GC activity except for *P. ginseng* and *Laserpitium zernyin* that are used to treat various illnesses including inflammation in eastern Asia and central and southern Europe, respectively [14, 16]. In these studies, the major compounds with dissociated GC activity were ginsenoside Rg1 and daucane esters (2β-angeloyloxy-10α-acetoxy-8-daucene-2,4,10-triol and vaginatin) in *P. ginseng* and *L. zernyin*, respectively.

A limitation of the present study is there is scope for additional investigations to confirm the reported findings. First, it is unclear which component in SK mediates the dissociated GC activity. We did not isolate a single active component from SK in this study, and therefore, bioassay-guided fractionation should be conducted to identify the active components in our future studies. Second, although we confirmed the in vitro dissociated GC activities of SK, the in vivo anti-inflammatory effects of active components of SK should be verified in comparison with HC. Lastly, we did not rule out the induction of other adverse effects of GCs such as wound healing inhibition and osteoporosis by SEE, which should be investigated in future studies.

## Conclusions

To the best of our knowledge, this is the first study to report that the SEE exhibits dissociated GC activity. Furthermore, SEE has the potential to be a better therapeutic agent than HC because it exhibits sufficient transrepression with IL-6 inhibition and less transactivation, which induces adipogenesis. We believe that some components in SK can be identified as potential dissociated GCs to treat inflammatory disorders through further studies.

## Data Availability

The datasets used and/or analyzed during the current study are available from the corresponding author on reasonable request.

## Abbreviations

GC: Glucocorticoid
GR: GC receptor
GRE: GC response element
SK: *Salsola komarovii*
SEE: SK ethanolic extract
NF: Nuclear factor
LPS: Lipopolysaccharide
IL: Interleukin
HC: Hydrocortisone
TNF-α: Human tumor necrosis factor-α
IBMX: 3-isobutyl-1-methylxanthine
FBS: Fetal bovine serum
DMEM: Dulbecco’s modified Eagle’s medium
FP: Fluorescence polarization
DPBS: Dulbecco’s phosphate-buffered saline
hsp: Heat shock protein
